# Reprogramming of Mouse Calvarial Osteoblasts into Induced Pluripotent Stem Cells

**DOI:** 10.1155/2018/5280793

**Published:** 2018-03-12

**Authors:** Yinxiang Wang, Jessica Aijia Liu, Keith K. H. Leung, Mai Har Sham, Danny Chan, Kathryn S. E. Cheah, Martin Cheung

**Affiliations:** ^1^Department of Biochemistry, Li Ka Shing Faculty of Medicine, The University of Hong Kong, Pokfulam, Hong Kong; ^2^Department of Anatomy, Li Ka Shing Faculty of Medicine, The University of Hong Kong, Pokfulam, Hong Kong

## Abstract

Previous studies have demonstrated the ability of reprogramming endochondral bone into induced pluripotent stem (iPS) cells, but whether similar phenomenon occurs in intramembranous bone remains to be determined. Here we adopted fluorescence-activated cell sorting-based strategy to isolate homogenous population of intramembranous calvarial osteoblasts from newborn transgenic mice carrying both *Osx1-GFP::Cre* and *Oct4-EGFP* transgenes. Following retroviral transduction of Yamanaka factors (Oct4, Sox2, Klf4, and c-Myc), enriched population of osteoblasts underwent silencing of Osx1-GFP::Cre expression at early stage of reprogramming followed by late activation of Oct4-EGFP expression in the resulting iPS cells. These osteoblast-derived iPS cells exhibited gene expression profiles akin to embryonic stem cells and were pluripotent as demonstrated by their ability to form teratomas comprising tissues from all germ layers and also contribute to tail tissue in chimera embryos. These data demonstrate that iPS cells can be generated from intramembranous osteoblasts.

## 1. Introduction

Bone constitutes a major part of the skeletal system that provides support and physical protection to various organs of our body. During development, embryonic stem cells (ESCs) give rise to three germ layers in which the mesoderm is a major source of the mesenchymal precursors giving rise to most of the bony skeleton via the formation of cartilage intermediate in a process called endochondral ossification. In contrast, intramembranous ossification involves direct conversion of mesenchymal tissue into bone and primarily contributes to the formation of the skull bones [1]. However, these undifferentiated mesenchyme cells are originated from cranial neural crest cells, which are ectomesenchymal cells arising from the crests of the neural folds. After delamination from the neural folds, cranial neural crest-derived mesenchyme cells migrate to the destined regions where they undergo condensation to produce osteoblasts, committed bone precursor cells [2]. The osteoblasts are responsible for the formation, deposition, and mineralization of the bone extracellular matrix. Extrinsic and intrinsic regulators have been defined to regulate different stages of osteoblast development from its initial specification to the production and calcification of bone matrix [3]. These studies provide important insight into the key molecules for the formation of bone tissue during development and also derivation of osteoblasts from various cell sources for therapeutic treatment of bone defects.

Although bone possesses cell intrinsic capacity to regenerate, minor injury, aging, or trauma always results in significant bone loss that precludes natural replacement of bone tissue. This can be resolved by autologous bone graft using patient's own healthy bone to replace missing bone, but this surgical procedure is always associated with severe pain at the site of removal and donor site morbidity [4]. In addition, allogenic bone grafts carry the potential risks of pathogen transmission from donor to recipient and immune rejection [5]. Adult bone marrow-derived mesenchymal stem cells (MSCs) provide a promising cell source for bone regeneration because of their inherent capacity to differentiate into an osteogenic lineage as well as potent paracrine anti-inflammatory properties [6]. However, the use of MSCs in bone regeneration may be limited by their extreme low yield (typically 0.001%–0.01%) obtained from bone marrow aspirates and their proliferative potential, which significantly decreases with age [7]. These significant limitations can be resolved by transcription factor-mediated reprogramming of embryonic skin fibroblasts into patient-specific induced pluripotent stem (iPS) cells [8], which have been shown to provide unlimited source of MSCs for the generation of functional osteoblasts both in vitro and in vivo [9]. Subsequent studies further revealed that bone marrow cells [10], adult liver and stomach cells [11], pancreatic cells [12], adult neural stem cells [13], and mature B lymphocytes [14], keratinocytes [15], and blood cells [16] can also be reprogrammed into iPS cells. It is tempting to speculate that these iPS cells derived from various cell sources could be differentiated into osteoblasts under appropriate culture conditions [9, 17–19]. Therefore, different lineages either at their progenitor or at terminally differentiated state can be subjected to cell fate conversion into pluripotent cells. Consistent with this notion, a recent report added human osteoblasts (hOBs) as an additional source of cells to be reprogrammed into iPS cells which differentiated into ectodermal and mesodermal cells but exhibited low capacity to form endodermal cells upon cultured in differentiation medium [20]. In this study, hOBs were extracted from iliac crest bone, which is generated by endochondral ossification. Whether intramembranous bone could also be reprogrammed into iPS cells remains to be determined. In addition, this study did not demonstrate the stepwise reprogramming process from osteoblasts into iPS cells and the pluripotent potential of hOB-iPS cells in vivo.

Here we isolated a pure population of osteoblasts from intramembranous bone of neonatal mice carrying both *Osx1-GFP::Cre* and *Oct4-EGFP* transgenes. Daily monitoring of the reprogramming process revealed initial silencing of *Osx1-GFP::Cre* transgene expression followed by late activation of *Oct4-EGFP* expression when iPS cells were formed with comparable reprogramming efficiency as skin fibroblast-derived iPS cells. These osteoblast-derived iPS (O-iPS) cells exhibited gene expression profiles similar to embryonic stem cells and formed teratomas comprising all three germ layers after subcutaneous transplantation into nude mice. Importantly, O-iPS cells gave rise to tissues that were incorporated into the chimera embryos. Altogether, these findings demonstrate that osteoblasts derived from intramembranous bone can be reprogrammed into iPS cells, which exhibit embryonic stem cell gene expression signatures and features of pluripotency.

## 2. Materials and Methods

### 2.1. Mice and Fibroblasts

Mice were maintained in the Laboratory Animal Unit of the University of Hong Kong. All mouse studies were approved by the Committee on the Use of Live Animals in Teaching and Research (CULATR) and were done in accordance with institutional and international standards and regulations. Osteoblasts were isolated from calvariae of newborn mice carrying both *Osx1-GFP::Cre* and *Oct4-EGFP*. *Oct4-EGFP* transgenic mice serve as control. The calvariae were digested with Dispase (Worthington Biochemical Co.) and Collagenase II (Sigma) at 37°C for 5 minutes with rocking. After centrifugation for 5 min at 2000 rpm, the cell pellet was resuspended in Dulbecco's modified Eagle's medium (DMEM, Gibco) plus 10% fetal bovine serum (BIOSERA) and seeded 1 × 10^6^ cells per 100 × 20 mm dish and cultured at 37°C.

Embryonic fibroblasts were isolated from the C57BL/6N mouse line derived from Charles River Lab, USA. The mouse embryos at embryonic day E13.5 were dissected from the uterus of a pregnant female mouse in 1× PBS, and heads and internal organs were removed. Each embryo was coarsely chopped by using a sterile razor blade and was digested with 0.25% trypsin (Invitrogen)/1 mM EDTA (USB) for 20 minutes at 37°C. The reaction was stopped by adding DMEM + 10% FBS, and the medium was replaced the next day. For dermal fibroblast, after harvesting calvarial from newborn mice, the dead newborn was washed with water once, then with 70% ethanol twice. Ethanol was completely removed. The mouse was cut on the dorsal side and along the length of the body with a scalpel such that the skin is cut but the internal parts of the body are intact. The skin was carefully separated from the rest of the viscera and was digested with 0.25% trypsin/1 mM EDTA overnight at 4°C. Forceps were used to separate the epidermis from the dermis, which is further cut into small pieces, and a sterile glass coverslip was placed over 10 skin pieces in the center of a 6-well plate. A few drops of fresh DMEM + 10% FBS medium were added into the space below the coverslip followed by the addition of fresh DMEM + 10% FBS medium to the well. Cells were cultured at 37°C for few days until confluency, and then the coverslips were removed.

### 2.2. Fluorescence-Activated Cell Sorting

Isolation of osteoblasts expressing GFP by fluorescence-activated cell sorting from four calvarial bones of newborn mice carrying Osx1-GFP::Cre and Oct4-EGFP was performed as described in Liu et al. [21]. Briefly, osteoblasts derived from transgenic strains were digested with 0.25% trypsin (Sigma)/1 mM EDTA (Thermo Fisher Scientific) for 10 minutes at 37°C, and the reaction was stopped by adding DMEM + 10% FBS. 2 × 10^6^ cells were pelleted and resuspended in fresh DMEM + 10% FBS. Wild-type osteoblasts were used for calibrating BD FACSAria I cell sorter before sorting GFP^+^ osteoblasts. The sorted GFP^+^ osteoblasts (1 × 10^4^ cells per well) were cultured in fresh DMEM + 10% FBS medium at 37°C.

### 2.3. Alizarin Red S Staining

Osteoblasts were fixed in 10% formaldehyde in 1× PBS for 15 mins, stained with 40 mM Alizarin Red S (Sigma) solution for 30 seconds to 5 minutes, and washed 3 times with distilled water to remove excess staining solution.

### 2.4. Quantitative RT-PCR

The expression levels of stem cell and various lineage maker genes were quantified by real-time RT-PCR. RNA was extracted from cells using RNAspin Mini Kit (GE Healthcare) following the manufacture's protocol. Total RNAs (2 *μ*g) were reverse-transcribed by using the Superscript III First-Strand Synthesis System (Invitrogen). 50 ng of cDNA with 0.2 *μ*M of each oligonucleotide primer was mixed with Power SYBR Green PCR Master Mix (Applied Biosystems) in a 25 *μ*l volume. The conditions were programmed as follows: initial denaturation: 95°C, 1 min followed by 40 cycles of 15 s stage at 95°C and 30 s at 60°C, then 15 s at 95°C, 1 min at 60°C, and 15 s at 95°C. All of the samples were triplicated, and level of transcripts from each gene was normalized to endogenous *Gapdh* control. Primer sequences are provided in Supplementary Table S1.

### 2.5. Generation of O-iPS and F-iPS Cells

10^5^ sorted cells were infected with retrovirus generated from Plat-E cells, which were transfected with pMXOct4 (id: 13366), pMXSox2 (id: 13367), pMXKlf4 (id: 13370), and pMXc-Myc (id: 13375) (Addgene) in 6-well dishes with 0.5 ml of each viral supernatant (total 2 ml per well), and spun at 2500 rpm at 20°C for 90 min. The reprogramming factor-infected osteoblasts were cultured in osteoblast medium for 5 days before replating 8 × 10^5^ cells per 10 cm dish precoated with mitomycin C-inactivated mouse embryonic fibroblast in ES maintenance media. Media were changed daily until ES-like colonies were observed. F-iPS cells were generated from dermal fibroblasts of newborn mice. 10^6^ fibroblast cells were plated onto all wells of a 6-well plate and spin-infected with the four viral supernatants. Cells were cultured further in DMEM media supplemented with 15% FBS, 1 × penicillin/streptomycin/glutamine (Invitrogen). On day 5, the cultured cells were trypsinized and replated in four 10 cm dishes on mitomycin C-inactivated mouse embryonic fibroblast with ES maintenance media. Media were changed every day until ES-like colonies were observed.

### 2.6. Immunofluorescence

iPS cells were grown on 8-well chamber slides. Cells were fixed in 4% paraformaldehyde (Sigma) for 10 min at room temperature (RT). Cells were washed with 2× PBS. Cells were permeabilized with 0.2% Triton X-100/PBS (PBST) for 10 min at RT and were incubated with blocking solution (1% BSA (USB)/PBST) for 30 min at RT. Appropriate primary antibody solution diluted in blocking solution was applied onto the slide and incubated overnight at 4°C. Cells were washed twice with PBS followed by incubating with appropriate secondary antibody diluted in blocking solution for 2 hours in darkness at RT. Cells were washed 3× with PBS. The last wash was aspirated, and a drop of VECTASHIELD® HardSet™ Mounting Medium with DAPI (Vector) was applied, and the slide was mounted with coverslip.

### 2.7. Genotyping of O-iPS Colonies

After being digested with 0.25% trypsin/1 mM EDTA, iPS cells were pelleted, resuspended, and digested in 100 *μ*l 1×TNE supplemented with 0.2 mg/ml Proteinase K for 16 h at 65°C. Then equal volume of phenol/isoamyl alcohol/chloroform (25 : 1 : 24, USB) was added into the mixture and centrifuged at full speed for 10 min. The upper aqueous phase was transferred to a 1.5 ml fresh eppendorf tube. One milliliter of prechilled absolute ethanol was added into the solution and centrifuged at full speed for 10 min. The DNA pellet was washed in cold 70% ethanol followed by centrifugation to remove the ethanol. After 10 min air dry, the pellet was resuspended in 20 *μ*l ddH_2_O.

### 2.8. Teratoma and Chimera Analysis

Prior to transplantation, O-iPS colonies were dissociated into single cell suspensions to enable transplantation of defined numbers of cells. Teratomas were assessed by injecting 10^6^ cells subcutaneously into the dorsal flank of nude mice, and teratoma formation was monitored for 4 weeks after injection. Collected tumors were processed for hematoxylin and eosin (H&E) staining. Chimera analysis was conducted by injecting Oct4-EGFP^+^ O-iPS cells into blastocysts isolated from C57BL/6 embryos, which were collected at the two-cell stage. The fertilized embryo was collected from the oviduct and cultured in KSOM media. The reconstituted blastocysts were implanted into 2.5-day pseudopregnant ICR females. Embryos were harvested at E17.5, and their tails were subjected to PCR genotyping with primers specific for Oct4-EGFP, Cre, and retroviral vectors carrying *Oct4*, *Sox2*, and *Klf4* genes.

### 2.9. Statistical Analysis

All analyses were performed in triplicate for *n* = 3 at least. Real-time RT-PCR was also performed at least 3 times. Data are expressed as average ±SEM, and the statistical significance (*p* value) was determined by a two-tailed Student's *t*-test using GraphPad Prism 6.

## 3. Results

### 3.1. Isolation and Characterization of Osteoblasts from Calvarial Bone

**Figure 1:**
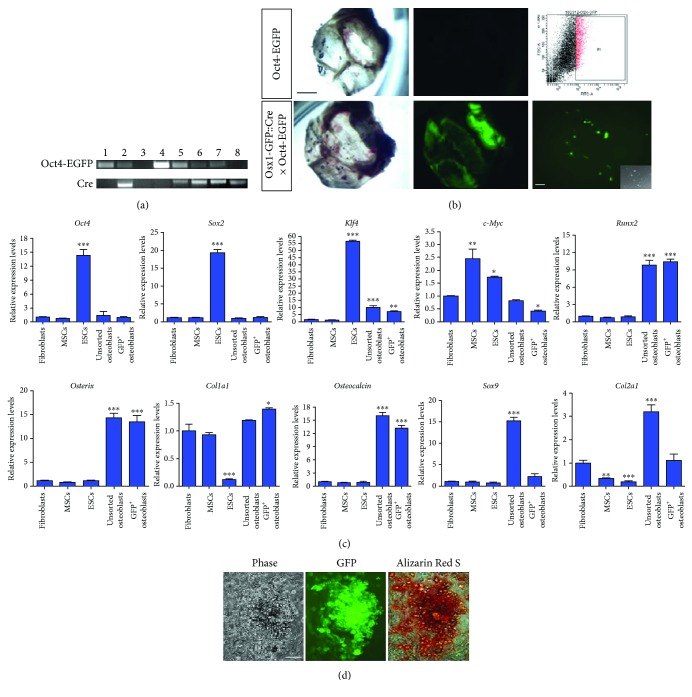
Isolation and characterization of calvaria-derived osteoblasts from newborn mice carrying both *Osx1-Cre::GFP* and *Oct4-EGFP* transgenes. (a) Genotyping analysis of 8 neonatal mice carrying *Osx1-GFP::Cre* and *Oct4-EGFP* transgenes for coding regions of *Oct4-EGFP* and *Cre*. (b) Bright field images of the newborn calvaria. No GFP expression was detected in *Oct4-EGFP* calvaria, whereas GFP expression was observed in calvaria isolated from a transgenic mouse carrying both *Osx1-GFP::Cre* and *Oct4-EGFP*. Representative FACS plot of isolating osteoblasts expressing GFP from calvarial bone. Sorted osteoblasts express GFP in culture. Inset shows phase image of sorted osteoblasts. Scale bars: 20 *μ*m for calvarial bone; 5 *μ*m for GFP^+^ cells. (c) qPCR analysis of the indicated transcript levels in MSCs, ESCs, unsorted osteoblasts, and sorted GFP^+^ osteoblasts. Individual mRNA expression levels were normalized to *Gapdh* with fold change relative to dermal fibroblasts, which is arbitrary set to 1. (d) From left to right: phase image of sorted osteoblasts. GFP^+^ osteoblasts were positive for Alizarin Red S staining. Scale bar: 20 *μ*m. Three independent experiments are represented in (c). Data are expressed as means ± SEM. ^∗^ *p* < 0.05; ^∗∗^ *p* < 0.01; ^∗∗∗^ *p* < 0.001.

To isolate an enriched population of osteoblasts for iPS reprogramming, we crossed *Osx1-GFP::Cre* with *Oct4-EGFP* transgenic mouse lines [22, 23] and analyzed 4 out of 8 neonatal mice that showed the presence of both *Oct4-EGFP* and *Cre* transgenes in their genome after genotyping analysis (Figure 1(a)). *Osx1-GFP::Cre* is a BAC transgenic mouse line in which expression of a Tet-off regulatable GFP::Cre fusion protein is placed under the transcriptional regulation of the *Osx1* promoter that is active in osteoblast precursor cells [22]. Oct4 is an important marker of the undifferentiated state and a central regulator of pluripotency in embryonic stem cells (ESCs) [24–26]. Therefore, *Oct4-EGFP* transgenic mouse served as a reporter for the formation of iPSCs, which express EGFP under control of an *Oct4* 18 kb genomic fragment containing the minimal promoter and proximal and distal enhancer [23]. We first isolated osteoblasts expressing GFP by fluorescence-activated cell sorting (FACS) from 4 calvarial bone of newborn mice harboring both transgenes, whereas no GFP expression was observed in calvarial bone derived from *Oct4-EGFP* transgenic mice. Upon plating, more than 90% osteoblasts showed GFP expression (Figure 1(b)). qPCR analyses revealed that GFP^+^ osteoblasts expressed high levels of characteristic markers *Runx2*, *Osterix*, *Col1a1*, and *osteocalcin* and low levels of *Klf4* and *c-Myc* transcripts whereas expression of markers for chondrocytes (*Sox9* and *Col2a1*) and ESC markers (*Oct4*, *Sox2*, and *c-Myc*) were low. In contrast, unsorted osteoblasts expressed *Sox9* and *Col2a1* in addition to osteoblast markers (Figure 1(c)). In addition, the majority of sorted GFP^+^ cells were positive for Alizarin Red S staining indicating that they are functional osteoblasts undergoing mineralization (Figure 1(d)). These results indicate that sorted GFP^+^ cells derived from calvarial bone contain homogenous population of functional osteoblasts without containing other cell types such as chondrocytes and ESCs.

### 3.2. Reprogramming of Osteoblasts into iPS Cells

**Figure 2:**
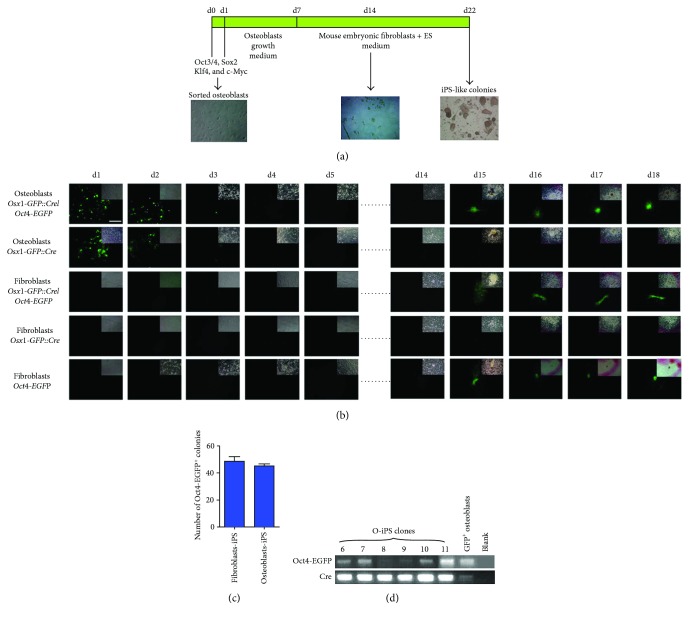
Reprogramming of osteoblasts into iPS cells. (a) Schematic illustration of iPS reprogramming strategy from sorted osteoblasts. (b) Phase (insets) and fluorescence images of osteoblasts and fibroblasts derived from transgenic mice carrying *Osx1-GFP::Cre* and/or *Oct4-EGFP* as well as their respective iPS cells at the indicated day (d) posttransduction. Scale bar: 5 *μ*m. (c) Quantification of the number of Oct4-EGFP^+^ F-iPS and O-iPS colonies formed. *n* = 3. Data are expressed as means ± SEM. (d) Genotyping of 6 O-iPS colonies for the presence of *Oct4-EGFP* and *Cre* transgenes. Sorted GFP^+^ osteoblasts serve as a positive control and blank as a negative control.

Since sorted osteoblasts expressed low levels of *Klf4* and *c-My*c that might not be sufficient to induce iPS reprogramming with other Yamanaka factors (Sox2 and Oct4), we used all four reprogramming factors to transduce 4 × 10^4^ GFP^+^ osteoblasts using retroviruses. After culture of retroviral-transduced osteoblasts in growth medium for 6 days, they were transferred and cultivated onto the feeder layer comprising mouse embryonic fibroblasts in ES medium and daily monitored for the appearance of Oct4-EGFP^+^ ES-like colonies. We observed iPS-like colonies on day 22 posttransduction, a time when iPS reprogramming is expected to be completed [27] (Figure 2(a)). Since somatic gene expression is subjected to epigenetic silencing during the course of iPS reprogramming [28], we anticipated that silencing of *Osx1* promoter driving GFP expression could occur prior to completion of reprogramming, which activates Oct4-EGFP expression in iPS cells. We used osteoblasts derived from *Osx1-GFP::Cre* transgenic mice without *Oct4-EGFP* transgene as a control to determine at what time point the GFP expression driven by *Osx1* promoter is silenced. In addition, skin fibroblasts isolated from single and double transgenic mice at newborn stage were used for comparing the efficiency of iPS cells generation with sorted osteoblasts. We found that GFP expression in osteoblasts derived from *Osx1-GFP::Cre*/*Oct4-EGFP* and *Osx1-GFP::Cre* transgenic mice was diminished on day 3 and completely lost on day 4 posttransduction, whereas GFP expression was only initiated and observed in iPS-like colonies harboring both transgenes from day 15 onward (Figure 2(b)), indicating that GFP expression detected was derived from activation of *Oct4-EGFP* transgene in iPS cells. While GFP expression was not detected in transduced fibroblasts derived from *Osx1-GFP::Cre*/*Oct4-EGFP* and *Osx1-GFP::Cre* transgenes as expected, we observed similar dynamics for the activation of GFP expression in fibroblast-derived iPS (F-iPS) cells carrying both transgenes or *Oct4-EGFP* alone as observed in osteoblasts (Figure 2(b)). Both osteoblast- and fibroblast-derived iPS cells exhibited comparable reprogramming efficiency (0.1075%/0.1175%; Figure 2(c)). To further examine whether iPS colonies were originated from *Osx1-GFP::Cre*/*Oct4-EGFP*-derived osteoblasts, we manually picked 6 individual colony to determine the presence of *Oct4-EGFP* and *Cre* transgenes in their genome by PCR. Sorted GFP^+^ osteoblasts and blank served as positive and negative control, respectively. Genotyping analysis showed that the coding regions of *Oct4-EGFP* and *Cre* were detectable in the genome of 6 iPS-like colonies (Figure 2(d)), confirming that they are originally derived from osteoblasts carrying both transgenes. We therefore named them as osteoblast-derived iPS (O-iPS) cells. Taken together, these findings suggest that loss of GFP expression in osteoblasts at the initial phase of iPS reprogramming could be due to epigenetic silencing of *Osx1* promoter activity by Yamanaka factors and subsequent emergence of GFP expression in O-iPS cells was derived from transcriptional activation of Oct4 promoter. Consistent with this notion, exogenous expression of four reprogramming factors was silenced or barely detectable in O-iPS clones indicating completion of iPS reprogramming (Figure S1).

### 3.3. Molecular Characterization of O-iPS Colonies

**Figure 3:**
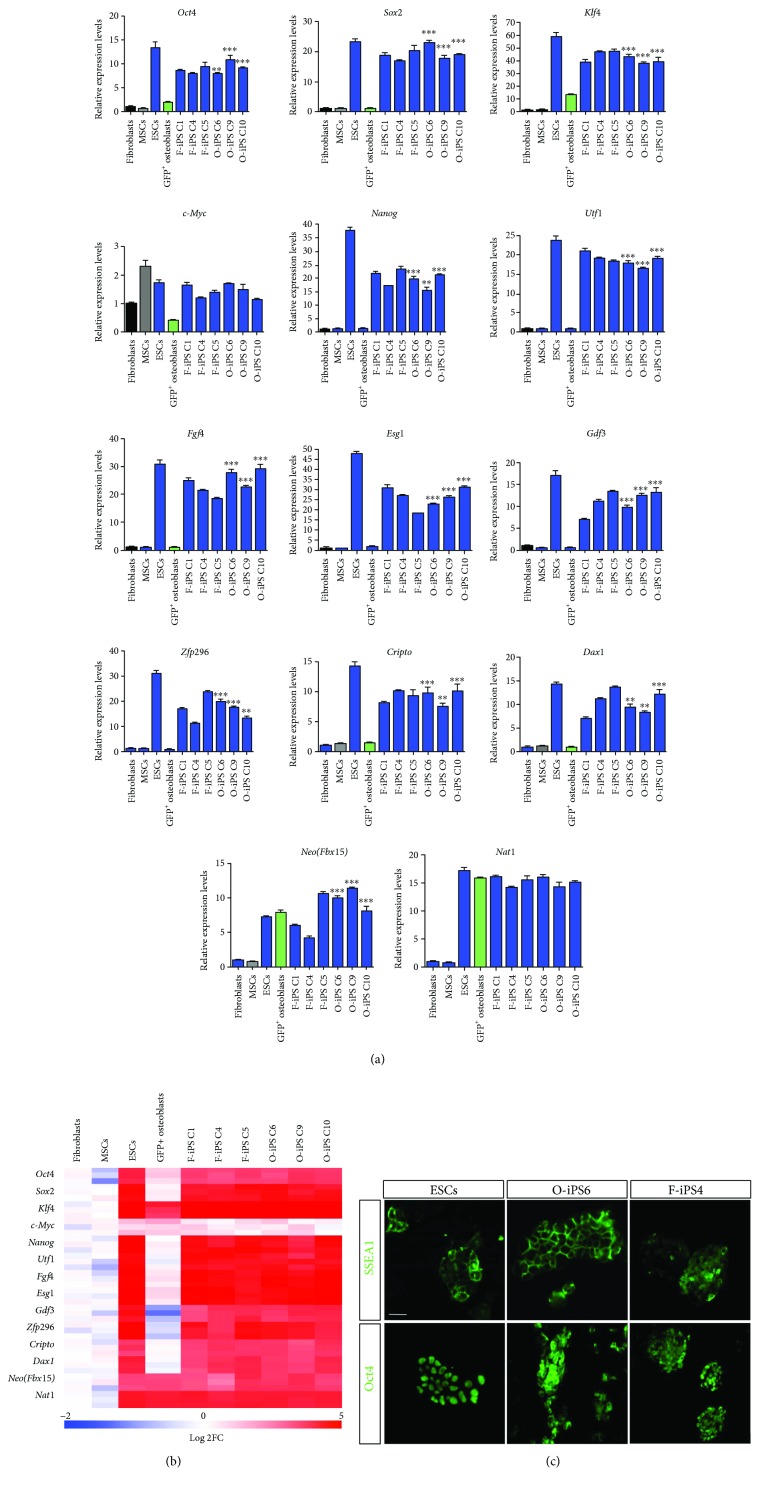
Gene expression profiles of O-iPS cells. (a) qPCR analysis for the indicted transcript levels in 3 F-iPS clones, 3 O-iPS clones, sorted GFP^+^ osteoblasts, ESCs, and MSCs. Individual mRNA expression levels were normalized to *Gapdh* with fold change relative to dermal fibroblasts which is arbitrary set to 1. (b) Heatmap of log fold change (Log2 FC) values for expression levels of the indicated transcripts in fibroblasts, MSCs, ESCs, GFP^+^ osteoblasts, F-iPS, and O-iPS clones. (c) Immunofluorescence of ESCs, O-iPS clone 6, and F-iPS clone 4 for antibodies against SSEA1 and OCT4. Scale bar: 100 *μ*m. Three independent experiments are represented in (a). Data are expressed as means ± SEM. ^∗∗^ *p* < 0.01; ^∗∗∗^ *p* < 0.001.

Previous studies showed that F-iPS colonies expressed ESC markers [8]. To examine whether O-iPS colonies also exhibited gene expression profiles characteristic of ESCs, we manually picked 3 O-iPS and 3 F-iPS colonies to compare their levels of ESC marker gene expression by qPCR. ESCs served as a positive control, whereas MSCs, fibroblasts, and GFP^+^ osteoblasts as negative controls. The results showed that 3 individual O-iPS clones expressed comparable levels of ES makers (*Oct4*, *Sox2*, *Klf4*, c-Myc, *Nanog*, *Utf1*, *Fgf4*, *Esg1*, *Gdf3*, *Zfp296*, *Cripto*, *Dax1*, *Neo*, and *Nat1*) with F-iPS clones and ESCs. Interestingly, we also detected significant levels of *Neo* and *Nat1* transcripts in GFP^+^ osteoblasts (Figures 3(a) and 3(b)). In addition, immunofluorescence showed that both O-iPS and F-iPS cells were positive for SSEA1 and OCT4 (Figure 3(c)). Consistent with previous observations [29], silencing retroviral-mediated expression of reprogramming factors correlated with the establishment of O-iPS cells (Figure S1). Collectively, these results demonstrate that like F-iPS, O-iPS clones also express signature genes characteristic of ESCs (Figure 3(b)).

### 3.4. Pluripotent Potential of O-iPS Cells

**Figure 4:**
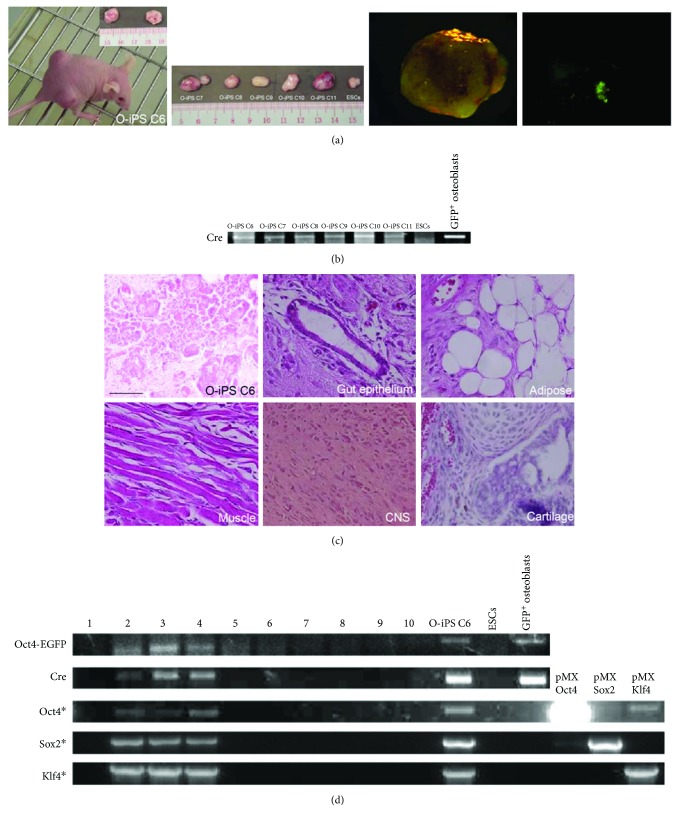
Pluripotent potential of O-iPS cells. (a) Formation of two teratomas derived from O-iPS6 cells under the skin in nude mice 4 weeks posttransplantation. Inset shows the size of teratomas harvested from nude mice. Teratomas derived from 5 different O-iPS cells and ESC-derived teratoma serves as a positive control. GFP expression was detected in an O-iPS6-derived teratoma. Scale bar: 1 cm. (b) Genotyping of teratoma tissues derived from 6 O-iPS colonies for *Cre* transgene. Sorted GFP^+^ osteoblasts serve as a positive control. (c) H&E staining of teratoma sections showed differentiation of O-iPS6 into various tissues from all three germ layers. CNS: central nervous system. Scale bar: 100 *μ*m. (d) Genotyping of coding regions for *Oct4-EGFP*, *Cre*, and integrated retroviral vectors carrying reprogramming factor genes (*Oct4*, *Sox2*, and *Klf4*) on tail tissues derived from E17.5 chimera embryos. ESCs serve as a negative control. Sorted GFP^+^ osteoblasts and retroviral vectors carrying reprogramming factors serve as positive controls.

To examine whether O-iPS colonies possessed pluripotent potential, we selected 6 O-iPS colonies (O-iPS C6, O-iPS C7, O-iPS C8, O-iPS C9, O-iPS C10, and O-iPS C11) and injected 10^6^ cells per clone subcutaneously into the dorsal flank of nude mice. After 4 weeks of injection, all of them were capable of forming teratomas in which small patches of GFP^+^ cells were observed indicating that O-iPS clone could differentiate into the osteoblastic lineage accompanied with activation of Osx1-GFP expression while Oct4-EGFP expression was silenced upon osteogenic differentiation (Figure 4(a)). Genotyping of *Cre* gene indicated that teratoma tissues were derived from O-iPS cells (Figure 4(b)). Detailed morphological analyses further revealed that teratoma was composed of tissues derived from all three germ layers including muscle, adipose, gut epithelial, and central nervous system (Figure 4(c)). Therefore, these teratoma studies demonstrate that O-iPS cells possessed pluripotent potential.

We then performed chimera assay to further interrogate the pluripotency of O-iPS clone. We selected O-iPS C6 clone for injection into ICR-derived blastocyst. Genotyping analysis of the DNA extracted from the tail of chimera embryos revealed that 3 out of 10 embryos at E17.5 harbored *Oct4-EGFP* and *Cre* transgenes as well as sequences of retroviral vector carrying *Oct4*, *Sox2*, and *Klf4* genes (Figure 4(d)). These results suggest that O-iPS C6 clone contributed to some tissue formation in the tails of chimera embryos. Collectively, our findings show that O-iPS cells exhibit pluripotent features with ability to form teratomas and chimera embryos.

## 4. Discussion

In 2006, Shinya Yamanaka announced a milestone finding that iPS cells can be derived from skin fibroblasts by retroviral-mediated expression of two pluripotent transcription factors, Oct4 and Sox2, and two proto-oncogenes, Klf4 and c-Myc (OSKM) [8]. These scientific breakthroughs have revolutionized our view about cellular plasticity that lineage committed or terminally differentiated cells can be reprogrammed into pluripotent state. This notion is further supported by numerous follow-up studies in which many different cell types from a wide range of species can be reprogrammed to pluripotency by ectopic expression of OSKM [30]. A recent report demonstrated that human osteoblasts derived from iliac crest bone, which is generated from endochondrial ossification, can be converted into iPS cells (hOB-iPSCs) [20]. In this study, osteoblasts were isolated as they emerged from the human bone chips in growth media. However, the homogeneity of osteoblast population extracted by this crude preparation remains questionable and the assessment of iPS cell identity was mainly based on cell morphology, markers analysis, and in vitro differentiation assays. Here we reported a more rigorous approach by taking advantage of mice carrying both *Osx1-GFP::Cre* and *Oct4-EGFP* transgenes as reporters that allowed us to isolate homogenous population of osteoblasts from calvarial intramembranous bone by FACS as evidenced by expression of characteristic osteoblast markers compared to unsorted population, which expressed chondrocyte makers Sox9 and Col2. In addition, the dynamics of reprogramming process from sorted osteoblasts to iPS cells can be monitored on a daily basis, and we observed a rapid silencing of Osx1-GFP expression after 3 days of reprogramming followed by late activation of pluripotent Oct4-EGFP expression. This is consistent with the idea that iPS reprogramming is a stepwise process involving transcriptional and epigenetic changes that cause downregulation of somatic gene expression (Osx1-GFP) and then transition to a state that is positive for the embryonic marker SSEA1 and, finally, induce the full pluripotency network (Oct4-EGFP) [31, 32]. Although the starting osteoblast population exhibited homogenous expression of Osx1-GFP, random integration of retroviruses results in different expression levels of each individual factor, which induced stochastic gene expression changes in the host cells that confer only a fraction of osteoblasts with the right levels of transgenes expression to proceed with the correct transition of reprogramming events [33]. Failure to transition through any one of these steps (e.g., unable to silence Osx1-GFP expression) would lead to a block in reprogramming that account for the low overall reprogramming efficiency. Consistent with this model, several intermediate cell types exist at different stages of reprogramming, and some of these intermediates are still plastic and can be reverted to the early reprogramming state even if they already express marker of pluripotency SSEA1 [34]. Further analysis of these intermediates revealed transient activation of genes associated with progenitor state of the starting cell population that follows a reversal of cell lineage development during iPS or lineage reprogramming [35]. Whether calvarial osteoblasts could be reprogrammed via neural crest lineage as intermediates before pluripotency remains to be determined. Nevertheless, successful O-iPS generation indicates that the stages of partial reprogrammed cells with transient fates have been passed that require continuous OSKM expression at the early stage and silencing of their expression later in reprogramming is a prerequisite for these cells to become more committed to the pluripotent state [32].

Previous in vitro studies showed that hOB-iPS cells were able to differentiate into ectodermal and mesodermal cell types but not endodermal lineage. In contrast, we provided in vivo evidence that O-iPS cells gave rise to teratomas containing tissues derived from all three embryonic germ layers including induction of osteoblast lineage and also contributed to some tissue formation in chimera embryos, indicating that O-iPS cells are pluripotent. Importantly, tissues from teratomas and chimera embryos carried both *Osx1-GFP::Cre* and *Oct4-GFP* transgenes integrated into the genome confirming of their osteoblast origin and also reflecting the power of our approach using transgenic mice to follow the progression of osteoblast reprogramming into iPS cells not only in vitro but also in vivo.

In summary, using *Osx1-GFP::Cre* and *Oct4-EGFP* transgenic mice, we demonstrate the ability to monitor stepwise reprogramming process from enriched population of intramembranous osteoblasts into iPS cells. These O-iPS cells exhibit stem cell-like gene expression signature and features of pluripotency. Considering neural crest origin of intramembranous osteoblasts, our study allows us to further investigate whether multipotent neural crest cells can be formed and enriched as intermediate during iPS reprogramming for therapeutic use in the future.

## Supplementary Materials

Supplementary 1 Figure S1: quantification of transcript levels for the endogenous and exogenous expression of reprogramming factors in O-iPS and F-iPS cells. qPCR analysis of endogenous (endo) and exogenous (exo) *Oct4*, *Sox2*, *Klf4*, and *c-Myc* transcript levels. Individual mRNA expression levels were normalized to *Gapdh* with fold change relative to dermal fibroblasts, which is arbitrary set to 1. *n* = 3. Data are expressed as means ± SEM. ^∗∗^ *p* < 0.01; ^∗∗∗^ *p* < 0.001.

Supplementary 2 Table S1: list of primers used for genotyping analysis and real-time RT-PCR amplification in this study.
