# Supplementary material for: Reprogramming of Mouse Calvarial Osteoblasts into Induced Pluripotent Stem Cells
Source: Stem Cells Int. 2018 Mar 12;2018:5280793. doi: 10.1155/2018/5280793 (PMC5867603; doi:10.1155/2018/5280793)
Supplement: Supplementary 2 — Table S1: list of primers used for genotyping analysis and real-time RT-PCR amplification in this study. [file 5280793.f2.doc]

**Supplementary Table S1. List of primers used for genotyping analysis and real time RT-PCR amplification in this study.**

| **Markers** | **Forward primer 5’-3’** | **Reverse primer 5’-3’** |
| --- | --- | --- |
| *Endo-Oct4* | TCTTTCCACCAGGCCCCCGGCTC | TGCGGGCGGACATGGGGAGATCC |
| *Endo-Sox2* | TAGAGCTAGACTCCGGGCGATGA | TTGCCTTAAACAAGACCACGAAA |
| *Endo-Klf4* | CCATCGGACCTACTTATCTGC | AAAACCTCAAACCAAAACCC |
| *Endo-c-Myc* | TGACCTAACTCGAGGAGGAGCTGGAATC | AGTTTGAGGCAGTTAAAATTATGGCTGAAGC |
| *Runx2* | GGAGCTCGGCGGAGTAGTTC | CTGTGGTTACCGTCATGGCC |
| *Osterix* | CGCTTTGTGCCTTTGAAAT | CCGTCAACGACGTTATGC |
| *Col1a1* | GCAACAGTCGCTTCACCTACA | CAATGTCCAAGGGAGCCACAT |
| *Osteocalacin* | CAGACACCATGAGGACCATC | GGACTGAGGCTCTGTGAGGT |
| *Sox9* | AGCTCACCAGACCCTGAGAA | TCCCAGCAATCGTTACCTTC |
| *Col2a1* | TTCCACTTCAGCTATGGCGAT | GACGTTAGCGGTGTTGGGAG |
| *Oct4-EGFP* | ctaggtgagccgtcttttca | TTCAGGGTCAGCTTGCCGTA |
| *Cre* | TGGATGCCACCTCTGATG | CTGCACACAGACAGGAGC |
| *Nanog* | AGGGTCTGCTACTGAGATGCT | CAACACCTGGTTTTTCTGCCACCG |
| *Utf1* | ACGTGGAGCATCTACGAGGT | TAGACTGGGAGTCGTTTCTG |
| *Fgf4* | CGTGGTGAGCATCTTCGGAGTGG | CCTTCTTGGTCCGCCCGTTCTTA |
| *Esg1* | GAAGTCTGGTTCCTTGGCAGGATG | ACTCGATACACTGGCCTAGC |
| *Gdf3* | GTTCCAACCTGTGCCTCGCGTCTT | AGCGAGGCATGGAGAGAGCGGAGCAG |
| *Zfp296* | CCATTAGGGGCCATCATCGCTTTC | CACTGCTCACTGGAGGGGGCTTGC |
| *Cripto* | ATGGACGCAACTGTGAACATGATGTTCGCA | CTTTGAGGTCCTGGTCCATCACGTGACCAT |
| *Dax1* | TGCTGCGGTCCAGGCCATCAAGAG | GGGCACTGTTCAGTTCAGCGGATC |
| *Neo (Fbx15)* | GCT ATT CGG CTA TGA CTG GGC ACA | CCA CCA TGA TAT TCG GCA AGC AGG |
| *Nat1* | ATTCTTCGTTGTCAAGCCGCCAAAGTGGAG | AGTTGTTTGCTGCGGAGTTGTCATCTCGTC |
| *exo-Oct4* | GGCTTCAGACTTCGCCTCC | AACCTGAGGTCCACAGTATGC |
| *exo-Klf4* | CCCAGTGTGGTGGTACGGGAAATC | GTCGTTGAACTCCTCGGTCT |
| *exo-Sox2* | GCGGAGTGGAAACTTTTGTCC | CGGGAAGCGTGTACTTATCCTT |
| *exo-c-Myc* | CCCAGTGTGGTGGTACGGGAAATC | GCTCGCTCTGCTGTTGCTGGTGATAG |
| *pMX-Oct4* | GACGGCATCGCAGCTTGGATACAC | AGTTGCTTTCCACTCGTGCT |
| *pMX-Sox2* | GACGGCATCGCAGCTTGGATACAC | TCTCGGTCTCGGACAAAAGT |
| *pMX-Klf4* | GACGGCATCGCAGCTTGGATACAC | GTCGTTGAACTCCTCGGTCT |
| *Gapdh* | AGGTCGGTGTGAACGGATTTG | TGTAGACCATGTAGTTGAGGTCA |

Endo: endogenous; exo: exogenous
